# Persistence of marine fish environmental DNA and the influence of sunlight

**DOI:** 10.1371/journal.pone.0185043

**Published:** 2017-09-15

**Authors:** Elizabeth A. Andruszkiewicz, Lauren M. Sassoubre, Alexandria B. Boehm

**Affiliations:** Department of Civil and Environmental Engineering, Stanford University, Stanford, CA, United States of America; University of Hyogo, JAPAN

## Abstract

Harnessing information encoded in environmental DNA (eDNA) in marine waters has the potential to revolutionize marine biomonitoring. Whether using organism-specific quantitative PCR assays or metabarcoding in conjunction with amplicon sequencing, scientists have illustrated that realistic organism censuses can be inferred from eDNA. The next step is establishing ways to link information obtained from eDNA analyses to actual organism abundance. This is only possible by understanding the processes that control eDNA concentrations. The present study uses mesocosm experiments to study the persistence of eDNA in marine waters and explore the role of sunlight in modulating eDNA persistence. We seeded solute-permeable dialysis bags with water containing indigenous eDNA and suspended them in a large tank containing seawater. Bags were subjected to two treatments: half the bags were suspended near the water surface where they received high doses of sunlight, and half at depth where they received lower doses of sunlight. Bags were destructively sampled over the course of 87 hours. eDNA was extracted from water samples and used as template for a *Scomber japonicus* qPCR assay and a marine fish-specific 12S rRNA PCR assay. The latter was subsequently sequenced using a metabarcoding approach. *S*. *japonicus* eDNA, as measured by qPCR, exhibited first order decay with a rate constant ~0.01 hr ^-1^ with no difference in decay rate constants between the two experimental treatments. eDNA metabarcoding identified 190 organizational taxonomic units (OTUs) assigned to varying taxonomic ranks. There was no difference in marine fish communities as measured by eDNA metabarcoding between the two experimental treatments, but there was an effect of time. Given the differences in UVA and UVB fluence received by the two experimental treatments, we conclude that sunlight is not the main driver of fish eDNA decay in the experiments. However, there are clearly temporal effects that need to be considered when interpreting information obtained using eDNA approaches.

## Introduction

Marine biodiversity is threatened by stressors including climate change, rising sea surface temperature, ocean acidification, overfishing, habitat loss, and nutrient, plastic, and pollution [1–9]. Biomonitoring, monitoring of organism abundance and diversity, is traditionally conducted using visual counts by divers or remote operated vehicles (ROVs), trawl nets, fishing, or tagging of individuals [10,11]. These traditional methods can disturb habitats and harm organisms [11–14] and resultant datasets are spatially and temporally sparse [15]. Researchers are exploring the use of a less-invasive method of biomonitoring which entails collecting water samples to capture extra-organismal, environmental DNA (eDNA) that has been shed from organisms and remains suspended in the water column [16–38]. eDNA from macroorganisms is in the form of scales, tissue, mucus, blood, feces, gametes, or any other secretion [35,37]. eDNA can be free-floating or bound to particles, with preliminary studies demonstrating the poly-disperse nature of eDNA [39–41]. Molecular methods are used to interrogate the eDNA. Quantitative PCR (qPCR) can be used to detect and quantify eDNA from specific organisms, or alternatively, eDNA metabarcoding can be used to obtain a census of organisms. In the eDNA metabarcoding approach, “universal” primers targeting a gene of interest are used in conjunction with next generation sequencing (NGS) [36]. Due to the ease of collecting water samples for eDNA analysis, temporally and spatially dense biological datasets are possible [37].

Numerous studies have demonstrated the use of eDNA for detecting macroorganisms in water [18,42–44]. However, there is uncertainty as to how these data relate to actual organism counts and their locations. The concentration of eDNA in water is controlled by eDNA sources (i.e., shedding) and sinks (i.e., decay) as well as its transport (i.e., advection, dispersion, settling, resuspension) [45]. A better understanding of these different processes will allow a link to be made between eDNA concentrations and organism abundance, and potentially bound the spatial and temporal range of where and when an organism shed the eDNA that was captured in the water sample.

The focus of the present study is eDNA decay in marine waters. Decay is defined as the disappearance of eDNA due to physical, chemical, or biological processes, and does not include settling under the influence of gravity. eDNA decay is expected to depend on a wide variety of factors. eDNA decay may depend on whether it is extra-cellular or cellular, or if it is particle-bound or free-floating. It may also depend on abiotic factors such as sunlight, water temperature, pH, and salinity [46], and biotic factors such as grazers or enzymes in the water column [46,47].

eDNA has been studied extensively in soils, sediments, and ice cores, and more commonly has been used to investigate microbial communities rather than macroorganism communities [48,49]. In those matrices, eDNA persistence depends on a variety of factors including soil type, depth of eDNA in the matrix, and soil chemistry, but it is accepted that eDNA can persist on the order of years in soils and sediments [32]. Recent persistence studies of macroorganism eDNA from fish and amphibians in freshwater indicate eDNA can persist from 4 to 52 days [44–47,50–54]. However, there are few studies that address the persistence of eDNA in marine waters, where abiotic and biotic stressors are expected to differ from freshwaters and sediments [37]. Furthermore, there are no studies that investigate how information on macroorganism presence/absence obtained from eDNA metabarcoding changes over time as a water parcel ages.

The present study investigates the decay of eDNA in oceanic waters. We use seawater from Monterey Bay mixed with aquarium water containing indigenous eDNA deployed in solute-permeable dialysis bags. Experiments were conducted under two different sunlight exposures to investigate the role of sunlight in controlling eDNA persistence. We chose to investigate sunlight specifically because it varies with depth in the water column as do fish populations, making it an important parameter for interpreting fish eDNA concentrations measured in marine waters. There is also a lack of studies investigating the effect of sunlight on eDNA decay in marine waters. We track changes in fish eDNA in the experiments using both qPCR and eDNA metabarcoding. Results provide insight into the persistence of eDNA and the effect of sunlight on the decay of eDNA in marine waters.

## Materials and methods

### Experimental design

**Fig 1:**
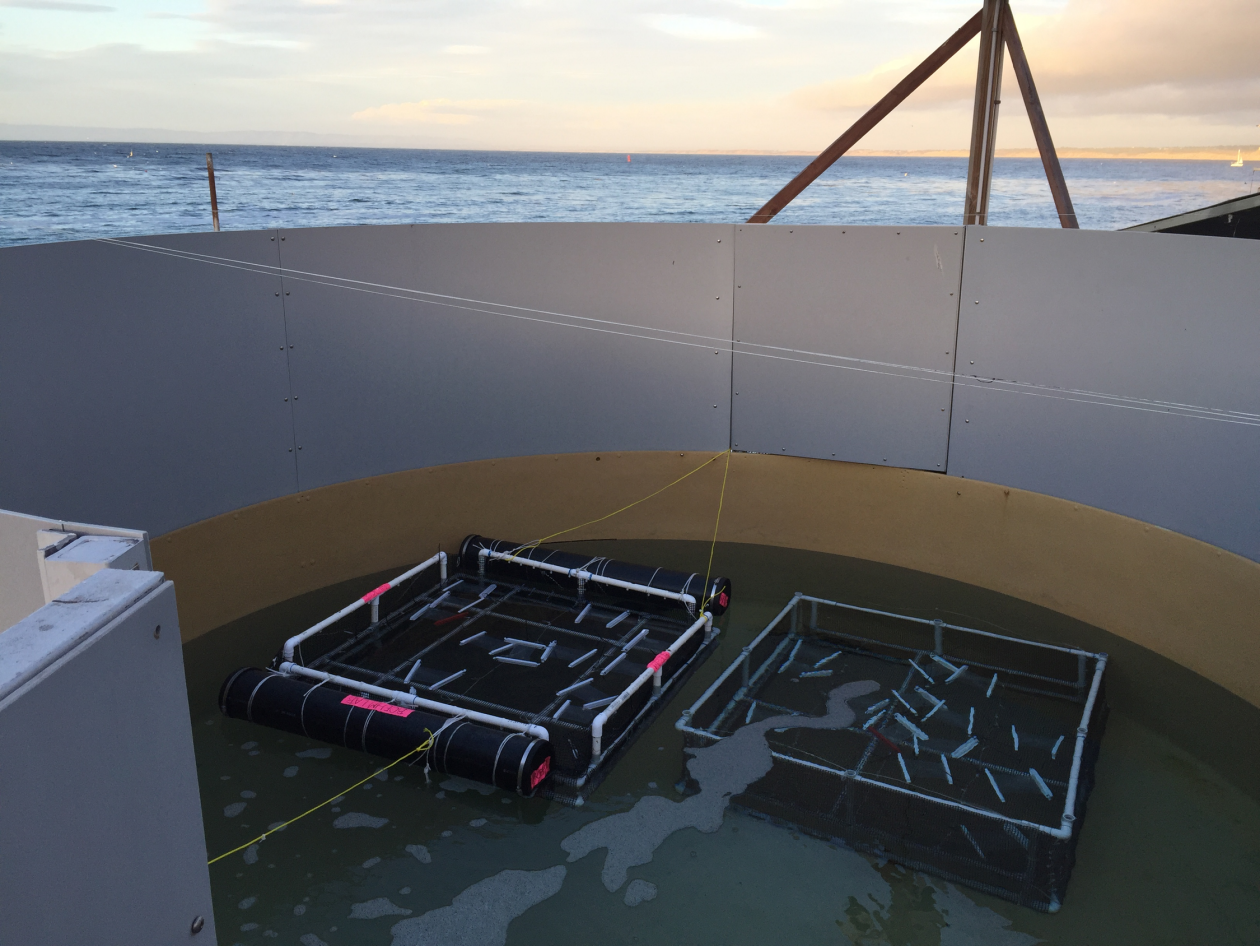
Deployment of dialysis bags in tank. 23 bags suspended at surface (middle of bags 5 cm beneath water surface) and 22 bags suspended at depth (middle of bags 70 cm beneath water surface).

Experiments were contained within dialysis bags (6–8 kDa molecular weight cutoff corresponding to 9–12 base pairs of double stranded DNA, 120 mm diameter Spectra/Por 1 RC Tubing, Spectrum Laboratories Inc., Rancho Domingo, CA) that were deployed in an outdoor seawater tank owned by the Monterey Bay Aquarium in Monterey, CA. The dialysis bags allowed passage of water and dissolved constituents through (therefore simulating more environmentally relevant conditions), but did not allow molecules or particles in or out of the bag larger than the pore size (i.e., target DNA). Maraccini et al. [55] determined the percent of transmittance of light (280–700 nm wavelength) through the dialysis bags. The tank held ~10,000 L of water sourced from Monterey Bay, but that had passed through a nominal pressure sand filter removing particles larger than ~20 μm and an aeration tower (hereafter this water is referred to as processed Monterey Bay seawater). The tank had walls surrounding it resulting in shading at certain times of day (Fig 1). Water flowed through the tank throughout the experiment at 0.0871 m^3^/min. The average temperature of the water over the course of the experiment (from data collected every minute at the closest sensor to the tank, provided by Monterey Bay Aquarium staff) was 16.8°C (standard deviation: 0.46°C; range: 15.8°C—17.8°C).

Dialysis bags were filled with 500 ml of a mix of 80% processed Monterey Bay seawater and 20% water from a tank located at the Tuna Research Conservation Center (TRCC) at Hopkins Marine Laboratory of Stanford University in Pacific Grove, CA. The water from the TRCC tank (hereafter referred to as TRCC tank water) was processed Monterey Bay seawater that had flowed through aquaria holding tuna (*Thunnus)*, Pacific Chub Mackerel (*Scomber japonicus*), and Pacific Sardines (*Sardinops sagax*) before it was sampled. These three species are also native to Monterey Bay. We opted to mix these two waters together to seed the dialysis bags at the start of the experiment in order to ensure we would have: (1) sufficient eDNA from *S*. *japonicus* in order to use a species-specific qPCR assay for quantification of decay rate constants, and (2) sufficient eDNA from a variety of marine macroorganisms to detect changes in marine fish communities over time via eDNA metabarcoding.

We filled 45 dialysis bags with the 80/20 mixture (hereafter referred to as T_0_ water). 23 bags were deployed at the surface of the water in the tank and 22 at the bottom of the tank. To hold all other variables constant and only test the effect of sunlight, we used the same tank for both treatments. We were limited by the depth of the tank and could not suspend the bags deep enough for complete darkness. We accounted for differences in UV exposure at depth in our analysis. Bags were deployed secured to a polyvinylchloride pipe frame with zip-ties used in a previous study [56] (Fig 1). The rig at the surface had buoys to keep the frame floating and the center of the bags was 5 cm beneath the water surface (hereafter referred to as surface). The second rig had no buoys and sank to the bottom of the tank, resulting in the dialysis bags being suspended 70 cm beneath the water surface (hereafter referred to as depth).

We destructively sampled triplicate bags (quadruplicate for some time points) to represent biological replicates both at the surface and depth approximately every 12 hours for 4 days (Table 1). The length of the experiment was chosen based by considering previous decay rate constants reported by others [45]. Experiments commenced on the evening of 16 October 2015 and ended the morning of 20 October 2015. Over the course of the 4 days, there were 2 rain events (resulting in <0.01 inches rain total) (overnight 18/19 October 2015 and early morning 19 October 2015, S1 Table).

**Table 1:** Sampling schedule.

Date	Time (PST)	Time Point	Time Since Start of Experiment (hh:mm)	Samples Collected (in triplicate unless noted)	Total # of Bags Sampled
10/16/15	16:30	T_0_	00:00	T_0_	3
10/17/15	7:15	T_1_	14:45	T1-S, T1-D	6
10/17/15	17:00	T_2_	24:30	T2-S, T2-D	6
10/18/15	7:10	T_3_	38:40	T3-S, T3-D	6
10/18/15	16:50	T_4_	48:20	T4-S, T4-D	6
10/19/15	7:30	T_5_	63:00	T5-S, T5-D	6
10/19/15	17:15	T_6_	72:45	T6-S*, T6-D	7
10/20/15	7:30	T_7_	87:00	T7-S*, T7-D*	8
					48

S indicates surface samples; D indicates depth samples;

* indicates quadruplicate samples taken

The absorbance of the ambient tank water was measured in triplicate using Uvikon XL UV-Vis Spectrophotometer (BioTek Instruments, Winooski, VT). The reference solution was deionized water and we used the average of the three wavelength scans. We used the Simple Model of the Atmospheric Radiative Transfer of Sunshine (SMARTS) model to obtain the intensity of light (280 nm– 700 nm) incident on the surface of the water for every 30 minutes on 18 October 2015 (midpoint of the study), which was used as a representative day for the whole experiment (S1 Text, S2 Table, S1 Fig). The SMARTS model does not account for cloud cover; the weather on 18 October 2015 was partly cloudy with scattered clouds for the majority of the day. The UVA+UVB (280–400 nm) and UVB (280–320 nm) range light intensity incident on the water in the bags was determined for bags deployed at the surface and at depth as described elsewhere [55]. In brief, we accounted for the absorbance of the water and the dialysis bag membrane material in the calculations, but did not account for potential cloud cover or shading from the walls of the tank. However, both treatments (surface and depth) experienced the same cloud cover conditions over the course of the experiment.

### Water filtration

We transported the sampled dialysis bags on ice to the laboratory for filtering. Each sample (n = 48) was vacuum-filtered through a 0.22 μm pore size (47 mm diameter) track-etched polycarbonate filters (Nucleopore Track-Etch Membrane, Whatman, GE Healthcare Bio-Sciences, Pittsburgh, PA) using 250 ml disposable analytical test filter funnels filled twice (ThermoScientific, Waltham, MA) to capture eDNA. Some samples (13 of 48) clogged the filters before all 500 ml could pass through, resulting in <500 ml of water filtered (S3 Table). Filtration blanks were created (n = 7) by filtering 50 ml of molecular-biology-grade water (Sigma Aldrich, St. Louis, MO) in the same manner as water samples to check for contamination during filtration. Filters were immediately placed in sterile 5 ml sterile polypropylene transport tubes, and stored at -20°C for the length of the experiment, and then transported back to Stanford University and stored at -80°C until extraction within 6 months of collection.

### Laboratory environment

DNA extraction and molecular work was performed at Stanford University. Benchtops were cleaned with 10% bleach for 10 minutes and then wiped with 70% ethanol. Benchtops were wiped with RNASE AWAY before beginning molecular work. Pipettes were wiped with RNASE AWAY and UV-irradiated for at least 10 minutes before use. DNA extractions were performed on one bench, PCR preparation was performed in a designated DNA-free hood, PCR amplification was performed in a separate room in the laboratory, and post-PCR work was performed in yet another separate room.

### DNA extraction and inhibition testing

DNA was extracted from the archived filters in 4 sets, adding in an extraction blank (n = 4, extraction reagents added to an empty 5 ml tube with no filter) for each set in addition to the experimental samples (n = 48) and filtration blanks (n = 7). Samples were randomized prior to extraction. We extracted DNA from each filter using the DNeasy Blood and Tissue Kit (Qiagen, Valencia, CA) with a modified lysis step (S1 Text). To increase DNA yield, we performed 2 elutions of 50 μL each for a total extract volume of 100 μL. We immediately quantified total DNA using a QUBIT fluorometer 2.0 (Life Technologies, Grand Island, NY) and stored extracts at -20^o^ C until amplification within 6 months of extraction. DNA extracts were then used for the following analyses: qPCR analysis using *S*. *japonicus* assay and mitochondrial 12S rRNA amplicon metabarcoding.

Before PCR or qPCR amplification, we tested a subset of DNA extracts for the presence of PCR inhibitors using serial dilutions (see S1 Text for methods and S2 Text for results) [57]. Based on the results of each inhibition test, we did not dilute DNA extracts for the *S*. *japonicus* qPCR amplification, but we did dilute extracts 1:10 before the conventional PCR amplification used for eDNA metabarcoding.

### *Scomber japonicus* qPCR assay

We used a recently published qPCR primer set and probe for Pacific Chub Mackerel (*S*. *japonicus*) targeting the cytochrome c oxidase subunit I (COI) gene [45]. The primers/probe sequences were: F 5’ GCTGAACAGTTTATCCTCCCCTCG 3’, R 5’ CCCAAGGATTGAGGAAACACCTGCTAG 3’, P 5’-FAM-TGGGAACCTGGCACACGCCGGG-BHQ. Each DNA extract was amplified in the following 20 μL reaction: Taqman Universal Mastermix II (1x), 0.2 mg/ml bovine serum album (BSA), forward and reverse primer (0.6 μM), probe (0.1 μM), 2 μL of DNA extract, and molecular-biology-grade water (Sigma-Aldrich, St. Louis, MO). Cycle temperature parameters are given in Sassoubre et al. [45]; the initial step is 95°C for 10 min, followed by 40 cycles of 95°C for 15 s and 60°C for 1 min. The cycle quantification (Ct) threshold was set to 0.01. Each PCR plate included 3 no template controls (NTCs) with molecular grade water added to the reaction in lieu of DNA extract.

We used standards constructed from genomic DNA (gDNA) extracted from *S*. *japonicus* tissue using the Qiagen DNeasy Blood and Tissue Kit (Qiagen, Valencia, CA). We quantified DNA extracted using a QUBIT fluorometer 2.0 (Life Technologies, Grand Island, NY). We ran standards in triplicate along with samples in each PCR plate at the following concentrations: 200 pg per reaction, 20 pg per reaction, 2 pg per reaction, 0.2 pg per reaction, and 0.02 pg per reaction. Standard curve data were pooled and used together to create a regression of DNA concentration per reaction versus Ct to calculate concentrations of unknown samples. The concentrations of unknowns were converted from mass DNA per reaction to mass DNA per volume of water filtered (S3 Table) using dimensional analysis. We set the limit of quantification at the lowest concentration of a known standard that all three triplicates were consistently assigned a Ct value. Environmental samples with Ct values assigned higher than the average Ct value for the lowest reliably amplified standard were deemed as below the limit of quantification and removed from further analysis.

### Data analyses for qPCR results

Disappearance of *S*. *japonicus* eDNA in the dialysis bags was modeled using a first order decay model: d*C*/d*t* = -*kC*, where *C* is the concentration of *S*. *japonicus* eDNA in mass per volume of water filtered, *t* is time, and *k* is the first order decay rate constant in units of 1/time. To calculate the first order decay rate constant and its standard error (SE), we fit a straight line to ln(*C*/*C*_*0*_) versus time using linear regression in R [58]. We used the average of the concentrations of the T_0_ samples as C_0_. We performed a z-test with *α* = 0.05 to test the null hypothesis that the *S*. *japonicus* eDNA rate constant derived from samples at the surface is the same as the rate constant derived from samples collected at depth. The z-statistic was generated with the equation $z=\frac{k_{1}-k_{2}}{\sqrt{{SE}_{1}^{2}+{SE}_{2}^{2}}}$. If |*z*| > 1.96, the null hypothesis was rejected.

### eDNA metabarcoding

In addition to the dialysis bag and source water (T_0_) samples (n = 48), filtration blanks (n = 7), and extraction blanks (n = 4), we added two different positive controls in triplicate (n = 6) to the eDNA metabarcoding analysis. The two positive controls used were (1) genomic DNA extracted from swordfish tissue (*Xiphias gladius)* and (2) a mock community with equal mass per volume of DNA from 9 species of bony fishes (S4 Table). The mock community, and the methods used to create it, is described in more detail elsewhere [59].

We used a two-step PCR method [60] to amplify a fish-specific fragment of the 12S rRNA gene in the extracted eDNA as well as add a unique tag to each sample. The method is described in detail in Andruszkiewicz et al. [61]. Briefly, DNA extract (diluted 1:10, see “Inhibition testing”) from each sample (n = 65 from 48 environmental samples, 6 positive controls, 7 filter blanks, and 4 extraction blanks) was amplified with the published fish-specific primers targeting a hypervariable region of the mitochondrial DNA 12S rRNA gene [62]. The primer sequences were F-5’ GTCGGTAAAACTCGTGCCAGC and R-5’ CATAGTGGGGTATCTAATCCCAGTTTG, amplifying a *ca* 170 bp region. Thermal conditions for the first PCR amplification were 95°C for 5 min followed by 40 cycles of 95°C for 15 s, 55°C for 30 s and 72°C for 30 s. Each extract was amplified in triplicate using eight-strip PCR tubes with individual caps to prevent cross contamination. A no template control (NTC) using molecular-biology-grade water in lieu of DNA template was added (1 per extract as each has its own tagged primers) to monitor for contamination. The replicate products from the first PCR generated from each extract were pooled, visualized on a gel, bead-cleaned using the Agencourt AMPure XP bead system (Beckman Coulter, USA), and then used as template in a PCR that used the same primers listed above but with the addition of 6 bp tags on the 5’ ends of both the forward and reverse primer (S5 Table). Thermal conditions for the second PCR amplification were 95°C for 5 min followed by 20 cycles of 95°C for 15 s, 57°C for 30 s and 72°C for 30 s. Products from the second PCR were again pooled, visualized, bead-cleaned, and quantified using a QUBIT fluorometer 2.0 (Life Technologies, Grand Island, NY). Samples not showing amplification on the gel visualization after the second PCR amplification were not carried on to library preparation, with the exception of the negative controls (filter blanks, NTC, and extraction blanks), which were carried over despite the fact that none of them showed amplification based on gel-visualization. In total 44 of the 48 experimental samples, the 6 positive controls, the 7 filter blanks, the 4 extraction blanks, and 1 representative NTC (all of the NTCs combined) were prepared for sequencing (n = 62).

The tagged products from the second PCR amplification were combined into 3 pools by adding 50 ng of DNA from each amplicon. Then 250 ng of each of those 3 pools was used as input into the KAPA Hyper Prep Kit (KAPA Biosystems, Wilmington, MA) to create 3 libraries; each library had a unique Nextflex DNA barcode (BIOO Scientific, Austin, TX) ligated on during library preparation. The 3 libraries were then combined with an equal mass of eDNA (100 ng per library) into a single tube. The final concentration of the 3 combined libraries was 10.8 ng/μL. The size and concentration of the combined libraries was confirmed using a Bioanalyzer with High Sensitivity DNA assay (Agilent Technologies, Santa Clara, CA) before sequencing on an Illumina MiSeq platform at the Stanford Functional Genomics Facility (2x250 paired-end sequencing with a 20% Phi-X spike-in control).

### Bioinformatics and statistical testing

Bioinformatic analyses were conducted within a Unix shell script described in detail elsewhere [60]. Briefly, we merged paired end reads, quality filtered, demultiplexed tagged reads, removed primers, and clustered sequences into OTUs with a cluster radius of 1 (S1 Text). A representative sequence from each OTU was compared to sequences deposited in the National Center for Biotechnology Information (NCBI) nucleotide (nt) database (downloaded 4 January 2017) using BLAST+ (2.2.31+) [63] to annotate OTUs. The following parameters were used: percent identity = 97%, word size = 30, e value = 1e-20. The authors who developed the MiFish-U primers as well as other published marine metabarcoding studies [62,64] use a 97% identity cut-off, justifying this choice. We used the “taxize” package in R [65] to summarize the BLAST+ results and annotate OTUs to the entry with the lowest e-value and highest percent identity to assign taxonomy. If multiple entries in the nt database matched an OTU with the same percent identity and e-value, we used the lowest common taxonomic rank to annotate the OTU. We removed reads from OTUs annotated as non-vertebrates and non-marine vertebrates. Experimental samples and positive controls were then rarefied to 40,000 reads using the “rrarefy” function in the R package vegan [66] in order to account for unequal sequencing depths; this number appears to be sufficient based on the rarefaction curve (S2 Fig).

We modeled the presence of genera identified using eDNA metabarcoding using a binary logistic generalized estimating equation (GEE). A GEE was required because genera presence was a repeated measure over time. The GEE was used to test two null hypotheses: (1) the odds of the eight genera being present does not change over time, regardless of sampling depth; and (2) the depth treatment (surface versus depth) does not affect the rate of genera disappearance [67]. The eight genera were defined as clusters and the model produced estimates of population-averaged trends rather than trends for any one genus. If a genus was present in at least one of the biological replicate samples at a given time point, we defined is as present; otherwise, it was absent. We implemented the GEE in R using the gee package [68] and set the correlation structure as exchangeable. Sampling depth (binary), time (continuous), and the interaction between sampling depth and time were predictors in the model. Time was converted to units of days to facilitate interpretation of the model coefficients. The assumption of the logit varying linearly over time was evaluated graphically. Robust standard errors and z-values were used to evaluate statistical significance of model coefficients, with p < 0.05 defined as significant.

We used a Mantel test (implemented using the vegan package in R) to investigate the relationship between vertebrate marine community composition and time. The similarity matrix for the vertebrate community was constructed using the Jaccard distance between each sample; the temporal distance matrix was calculated using the difference between sampling times. Finally, we investigated if community composition at each time was affected by sampling depth using an ANOSIM (implemented using the vegan package in R) with the Jaccard distance matrix and sampling depth as the factor. We considered results statistically significant if p <0.05.

## Results

### *Scomber japonicus* eDNA decay

**Fig 2:**
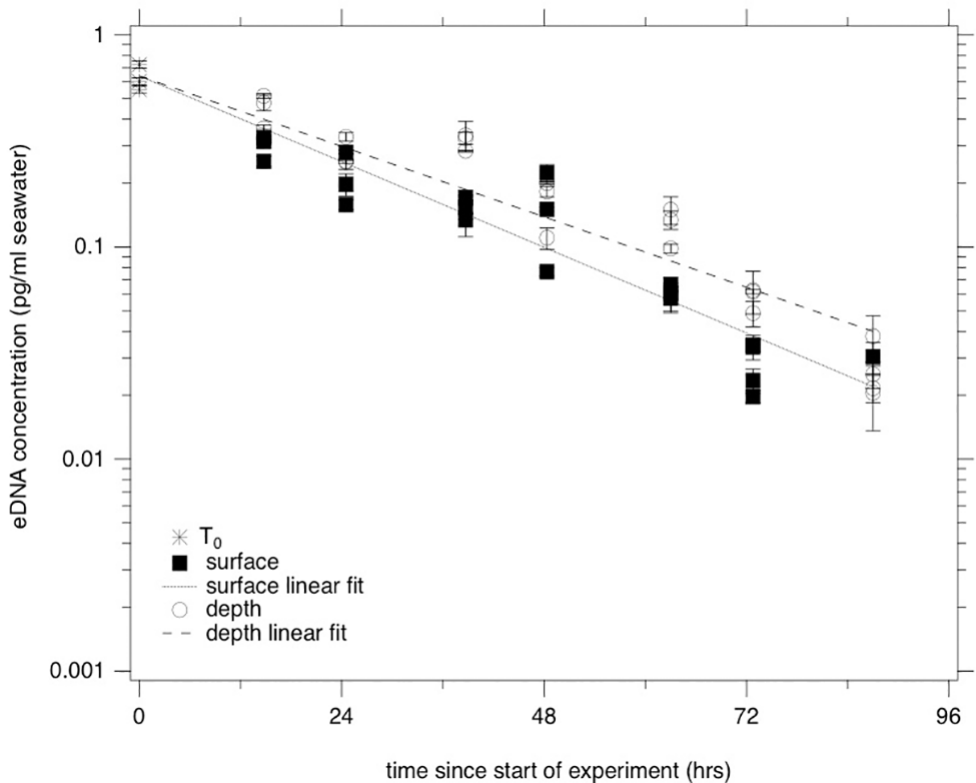
*S*. *japonicus* eDNA concentration (pg/ml seawater) as a function of time (hours) at surface (solid squares) and depth (open circles). Error bars represent standard deviation of triplicate qPCR reactions; triplicate samples shown as separate symbols at each time point (4 replicates for T6 surface and T7 depth, 1 replicate for T7 surface). Some error bars are small and hidden by overlapping symbols.

The *S*. *japonicus* qPCR assay had an average efficiency of 92.7%, and a limit of quantification of 0.1 pg/μL of DNA extract. The average Ct for the lowest reliably amplified standard (0.2 pg per reaction) was 38.15. All extraction blanks, filter blanks, and NTCs had undetermined Ct values indicating no contamination. C_0_ was 0.63 pg DNA/ml seawater. *S*. *japonicus* eDNA suspended at the surface of the tank had a decay rate constant of 0.039 +/- 0.0031 hr ^-1^ (unadjusted R^2^ = 0.89) and *S*. *japonicus* eDNA suspended at depth had a decay rate constant of 0.038 +/- 0.0029 hr ^-1^ (unadjusted R^2^ = 0.90) (Fig 2). The null hypothesis that the bags at surface and depth have the same decay rate constant was not rejected using a z-test with *α* = 0.05 (|*z*| = 0.28). Based on our calculations (S1 Text, S1 Fig), the bags at the surface received 0.031 W/m^2^/day UVB and 3.8 W/m^2^/day UVA+UVB radiation, and the bags at depth received 0.0093 W/m^2^/day UVB and 1.6 W/m^2^/day UVA+UVB radiation.

### eDNA metabarcoding

The MiSeq run produced 14,928,120 reads across environmental samples and positive controls, of which 92.57% had a Q score ≥30. After merging paired end reads, quality filtering, removing tags and adapters, and removing singletons, 3,962,266 reads remained (median per sample: 85,578; range per sample: 44–345,762) in the 44 experimental samples. These reads clustered into 544 OTUs, of which 233 were annotated and 311 were not. Of the annotated OTUs, 23 OTUs were annotated to non-vertebrates (e.g., *Saccharophagus degradans*) or non-marine vertebrates (e.g., *Canis lupis* or *Homo sapiens*). After removing OTUs annotated to non-vertebrates or non-marine vertebrates, 3,961,832 sequences remained in the experimental samples (median per sample: 85,544; range per sample: 36–345,756) comprising 521 OTUs. Due to unequal sequencing depths, samples were rarefied to 40,000 reads. 39 of the 44 sequenced experimental samples had >40,000 reads. The 5 experimental samples with less than 40,000 reads (T7-DA, T7-DC, T7-DD, T7-SA, T7-SB, see S3 Table for naming convention) were removed from subsequent analyses.

Negative controls (filter blanks, extraction blanks, representative NTC) had a total of 6,574 sequencing reads before rarefaction (median: 84; range: 15–4,682). The majority of reads in the negative controls (86%) were annotated to *C*. *lupis*. After removing reads from OTUs assigned to non-vertebrates or non-marine vertebrates, only 534 reads remained in all of the negative controls (median per sample: 36; range per sample: 6–123). Because of the 3 orders of magnitude difference in number of reads in experimental samples and negative control samples after removing non-vertebrates and non-marine vertebrates, we conclude that our reads from our negative controls can be considered negligible.

The two positive controls (swordfish tissue and mock community) had only 1 read out of 240,000 (6 x 40,000) that was not from the gDNA used to make the controls (S4 Table). For the mock community, although equal mass of each of the 9 taxa DNA extracts was added (S4 Table), the relative proportions of sequencing reads assigned to each taxon were not equal (range: 0% for *Paralichthys* to 30% for *Seriola*; not including not annotated OTUs). Based on these results, we chose to examine the eDNA metabarcoding data in a binary (presence/absence of OTU) rather than quantitative manner.

After rarefaction and removal of data from positive controls, the 39 experimental samples had 1,560,000 reads (39 x 40,000) assigned to 464 OTUs (190 annotated, 274 not annotated). The 190 annotated OTUs were assigned to different taxonomic ranks: 47 received species level annotation, 140 received genus level annotation, and 3 received subfamily level annotation. Several OTUs were assigned to the same taxon; the 190 OTUs include annotations from 1 unique subfamily, 8 unique genera, and 12 unique species level annotations (S6 Table). For example, 86 unique OTUs were all assigned to the genus *Scomber*. The 190 annotated OTUs include 99% of rarefied reads, meaning that only 1% of reads were assigned to the 274 OTUs that were not annotated.

Four OTUs account for the majority of rarefied sequencing reads in the experimental samples. Together, they represent >97% of rarefied reads across all time points (range per sample: 90.3% - 99.9%). The four OTUs were among those receiving an annotation and were assigned to *Scomber*, *Sardinops*, *Sebastes*, and *Sebastes*, respectively (Table 2). The two OTUs annotated to *Sebastes* are distinct (i.e., they were separated as distinct during the OTU clustering process), but annotated by the software to the same genus. They may represent different species.

**Table 2:** Four most abundant OTUs with the rank of annotation, common name of annotation, and percent of total rarefied reads assigned across all time points.

Taxonomic Assignment	Taxonomic Level	Common Name	Percent of Total Rarefied Reads
*Scomber*	Genus	Mackerel	84.3
*Sardinops*	Genus	Sardine/Pilchard	10.6
*Sebastes*	Genus	Rockfish	2.03
*Sebastes*	Genus	Rockfish	1.00

**Fig 3:**
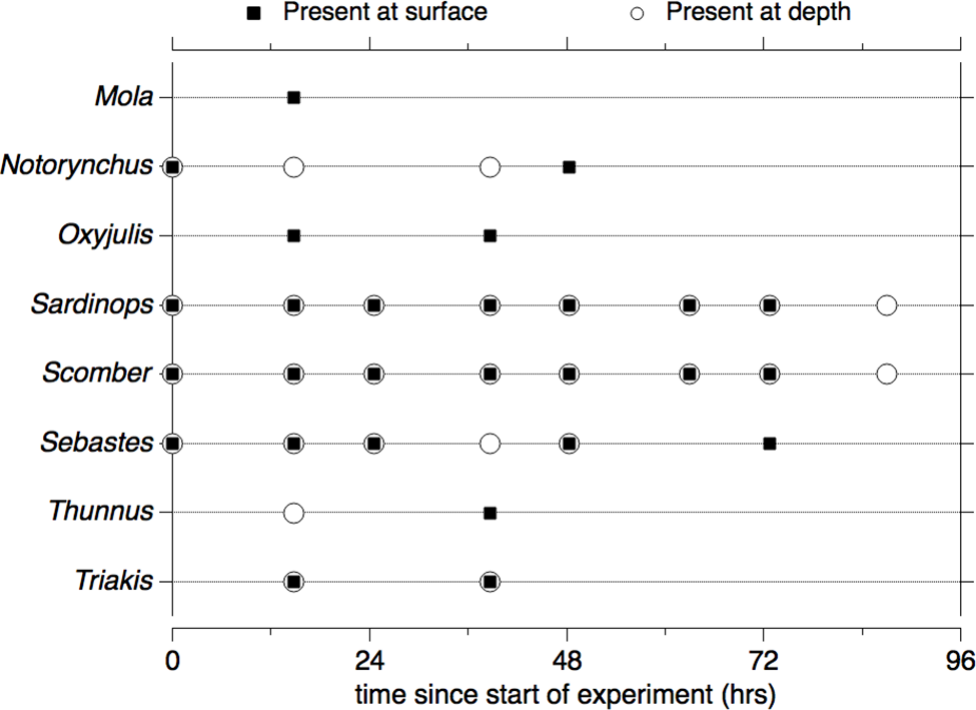
Genera identified as present using eDNA metabarcoding over the course of the experiment. Solid squares indicate presence of the genus in at least 1 biological replicate from surface samples; open circles indicate presence of the genus in at least 1 biological replicate from depth samples.

We examined how the detection of different genera varied over the time course of the experiment (see S2 Text and S3 Fig for the same analysis at the species level). This necessitated only considering OTUs annotated to at least the genus level (which meant we excluded 0.91% of the rarified reads, either assigned to the subfamily level or not annotated based on our criteria). There were 8 unique genera identified in our experiment over all time points. Some of these genera are present at each time point (considering surface and depth together, *Sardinops*, *Scomber*) while some are present at a subset of time points (Fig 3). The number of time points that each genus was detected at scales with the number of reads assigned to that genus at T_0_ (Spearman rho: n = 8, rho = 0.92, two-tailed p = 0.0014; Fig 3). All 8 genera were detected at T_1_ despite only 4 genera being detected at T_0_.

We used a GEE to investigate whether the presence of genera depended on the time since the start of the experiment, sampling depth of the experiment, or their interaction (whether the impact of time differed by sampling depth). We found no evidence to suggest that detection of genera depended on sampling depth at T_0_ (β = 0.054, p > 0.05). The interaction term between depth and time was not significant (β = -0.13, p > 0.05), which means that sampling depth did not statistically affect the rate of genera disappearance. Presence of genera was negatively associated with time (β = -0.35, p < 0.05); the corresponding odds ratios were 0.70 for surface and 0.62 for depth, indicating that the odds of genera being present decreased with an increase in time (Table 3). We also included a GEE model for the presence of species during the experiment, which produced the same results (S2 Text, S7 Table).

**Table 3:** Results of GEE model on presence of 8 genera over time.

	β	robust SE	robust z
(intercept)	0.52	0.43	1.2
sampling depth	0.054	0.42	0.13
time	-0.35	0.10	-3.4*
sampling depth:time	-0.13	0.18	-0.70

Dependent variable is presence of genera; sampling depth is a binary variable with values of 0 for surface (5 cm below water surface) or 1 for depth (70 cm below water surface); time is a continuous variable and is measured in days since the start of the experiment; sampling depth:time is the interaction term;

*indicates p > 0.05

We used a Mantel test to explore the association between fish community composition, as inferred from eDNA metabarcoding, and time. Fish community composition was more similar the closer samples were collected in time (r = 0.32, p = 0.001). When separated by surface samples and depth samples, the associations remained statistically significant (surface: r = 0.37, p = 0.001; depth: r = 0.38, p = 0.002). We found that there was no significant difference between community composition at a specific time between sampling depths using ANOSIM (R = 0.031, p = 0.13).

## Discussion

The concentration of *S*. *japonicus* eDNA, as measured by qPCR, declined over the duration of the experiment. Decay was first order with a rate constant of ~0.01 h^-1^ in both surface and depth treatments. This rate constant is of the same order of magnitude as that obtained by Sassoubre et al. [45], despite the difference in experimental design. Sassoubre et al. [45] quantified decay in a shaded, closed, batch system, whereas the present study quantified decay in a sunlit, open system where solutes could freely diffuse across dialysis membranes. The accordance of our decay rate constants and those of Sassoubre et al. [45], as well as the lack of difference between decay rate constants at surface and depth, suggest that sunlight was not important in controlling eDNA decay. This is further supported by the eDNA metabarcoding data; the odds of detecting genera were not affected by the depth of the experimental treatment and community composition at a specific time point did not vary with depth. Only one other study has looked at persistence of fish eDNA in marine water and quantified decay rates [69] but does not include any information on the mechanisms of decay.

The effect of sunlight on fish and amphibian eDNA decay in freshwater systems has been investigated in previous studies, but no clear result has been determined [51,52,54]. Strickler et al. [52] investigated bullfrog eDNA decay in freshwater and found no effect of sunlight. Pilliod et al. [51] found that salamander eDNA decayed faster in sunlit than in shaded freshwater but could not separate water temperature effects from sunlight effects. Finally, Merkes et al. [54] determined that there was no correlation between UV index and persistence in a study using silver carp eDNA in freshwater. No studies have investigated the impact of sunlight on decay of fish eDNA in marine waters. Over the course of our experiment, the surface treatment received >3 times more UVB energy and >2 times more UVA+UVB energy than the deep treatment. The lack of difference in decay rate constants despite the difference in UV energy implies that stressors other than sunlight likely contribute eDNA decay including bacteria, grazers and enzymes [46,47,53,70,71]. Future research should investigate these other mechanisms as well as the threshold at which eDNA decay would be impacted by UVA+UVB exposure for applications in other locations (near the equator for example) where solar intensity may be stronger.

The eDNA metabarcoding-derived census of fish was not stable over the course of the experiment. Fish community composition varied with time and the odds of detecting specific genera decreased with time. These results suggest that information on fish community composition gleaned from eDNA metabarcoding approaches may depend on how much time has elapsed since fish shed eDNA. This is likely due to the eDNA decaying over time, which affects its ability to be amplified by the primers and subsequently sequenced. These results, in combination with the decay observed in *S*. *japonicus* eDNA by qPCR, indicate that information obtained from fish eDNA may be best interpreted when additional information on eDNA age (time elapsed since eDNA shedding) is available. Further work to elucidate how various physical, chemical and biological processes affect the shedding, decay, advection and dispersion of eDNA in the ocean is needed to link eDNA measurements (from qPCR and metabarcoding) to actual fish numbers.

Our findings underscore that eDNA metabarcoding data obtained using the methods outlined herein should be interpreted in terms of presence/absence rather than quantitatively. The equal-mass of DNA from various fish in the mock community control did not yield equal proportion of reads assigned to each taxon in the mock community. Though we cannot speculate on why this occurs, some considerations are primer biases, PCR biases, and different sources of the eDNA in the sample [18,26,32,37,72,73]. Previous studies have shown correlation between biomass or population numbers and organism-specific eDNA concentrations as measured by qPCR [70,74–78], mostly in freshwater systems, and more recently a few studies suggest links between eDNA metabarcoding reads and fish abundance in aquatic systems [79,80]. However, other studies have not found a link between organism abundance and eDNA concentrations [81,82]. We decided not to compare the results for *Scomber* measured by qPCR and NGS in this paper because (1) the assays targeted different genes and (2) qPCR yields a concentration while NGS yields a proportion. We acknowledge the need for further research on linking qPCR concentrations and number of reads from eDNA metabarcoding. Our results indicate more research is needed to fully understand eDNA abundance and the connection to fish abundance, including time since shedding from an organism.

The present study used open, sunlit experimental systems to investigate the persistence of fish eDNA from Monterey Bay, California. We showed that over 86 hours, the information inferred from fish eDNA changes, in some cases substantially. This indicates that the time elapsed since eDNA was shed by organisms will be an important variable in linking data from eDNA to actual fish counts. Further work on establishing a modeling framework for interpreting information inferred from eDNA is needed.

## Supporting information

S1 Text Materials and methods supplement. Solar irradiance calculations, DNA extraction modifications, inhibition testing, and bioinformatic processing.
(DOCX)

S2 Text Results and discussion supplement. Results of inhibition testing for qPCR and conventional PCR, presence/absence of species-level annotations over the course of the experiment.
(DOCX)

S1 Fig Solar intensity versus time for 18 October 2015. Top panels are at 5 cm below water surface (black squares), bottom panels are at 70 cm below water surface (open circles). Left panels only account for UVB solar intensity (W/m^2^), right panels account for UVA+UVB solar intensity (W/m^2^).
(TIFF)

S2 Fig Rarefaction curves for experimental samples. Y-axis shows number of unique OTUs identified and x-axis shows number of reads included in the sample size.
(TIFF)

S3 Fig Species found in eDNA metabarcoding rarefied reads over time. Solid squares indicate presence of the species in at least 1 biological replicate from surface samples; open circles indicate presence of the species in at least 1 biological replicate from depth samples.
(TIFF)

S1 Table Weather over the course of the experiment. Obtained from the National Climatic Data Center's Climate Data Online (www.ncdc.noaa.gov); weather station located at Monterey Airport (36.588°N, -121.85°E), approximately 5 miles southeast of experiment tank. Time is in Pacific Standard Time (PST). Air temperature is measured in °C. Precipitation is measured in cm; T indicates "trace" precipitation, which means precipitation has been detected but insufficient for meaningful measurement.
(XLSX)

S2 Table Inputs to SMARTS model. (XLSX)

S3 Table Supplemental data sheet. Additional sampling information, naming convention, volume of water filtered, total number of sequencing reads.
(XLSX)

S4 Table Composition of mock community used as positive control and eDNA metabarcoding results of positive controls. List of 9 taxa from which extracts of gDNA were added in equal mass (200 ng) to the mock community. eDNA metabarcoding results of rarefied sequencing reads for the 3 replicates of the mock community and the 3 replicates of the swordfish gDNA extract included as positive controls. Colors indicate the 9 taxa included in the mock community and the different taxon found in the eDNA metabarcoding results corresponding to the 9 original taxa.
(XLSX)

S5 Table Sequences of tagged primers used for second PCR amplification. Tags with two samples amplified were run on separate libraries to distinguish samples.
(XLSX)

S6 Table Taxonomic ranks and annotations of identified annotated OTUs by eDNA metabarcoding in experimental samples after rarefaction. (XLSX)

S7 Table Results of GEE model on presence of 12 species over time. Dependent variable is presence of species. Sampling depth is a binary variable with values of 0 for surface (5 cm below water surface) or 1 for depth (70 cm below water surface). Time is a continuous variable and is measured in days since the start of the experiment. Sampling depth:time is the interaction term. * indicates p > 0.05.
(XLSX)
