# Supplementary material for: Persistence of marine fish environmental DNA and the influence of sunlight
Source: PLoS One. 2017 Sep 15;12(9):e0185043. doi: 10.1371/journal.pone.0185043 (PMC5600408; doi:10.1371/journal.pone.0185043)
Supplement: S1 Text — Solar irradiance calculations, DNA extraction modifications, inhibition testing, and bioinformatic processing. (DOCX) [file pone.0185043.s001.docx]

**S1 Text. Materials and methods supplement.**

**Solar irradiance calculations**

The output from the UV-Vis Spectrophotometer was absorbance (base 10). We then used the following equation to convert absorbance to the base e : a_e_ = ln(10)*a_10_ , where a_10_ is absorbance (base 10) and a_e_ is absorbance (base e). We then used the following equation: $\frac{I_{ave}}{I_{0}}=\frac{1-e^{-a_{e}\times l}}{a_{e}\times l}$ , where I_ave_ is the average intensity in suspension (W/m^2^), I_0_ is the intensity at surface (W/m^2^), a_e_ is absorbance to the base e, and l is the depth (cm). We corrected the depth using a pathlength correction factor $\frac{1}{cos\theta^{'}}$ where $\theta^{'}$ is the transmitted angle solved for using Snell’s Law and the following equation $\frac{n_{water}}{n_{air}}= \frac{sin\theta}{sin\theta^{'}}$ , where n is the index of refraction for air and water. We used 1.00 for n_air_ and 1.34 for n_water_. We obtained data from Weather Underground from a weather station located at the Monterey Airport approximately 5 miles southeast of the experimental set-up (S1 Table). For the SMARTS model inputs, we made similar assumptions to Maraccini et al. [1] (S2 Table). We used the SMARTS model to obtain solar intensity at the surface at each wavelength from 280 nm to 700 nm. We solved for the pathlength correction and the average intensity at each depth (5 cm below water surface and 70 cm below water surface) for each wavelength from 280 nm to 400 nm every 30 minutes from sunrise to sunset on 18 October 2015 as a representative day in the 4-day study. We summed the intensity from 280-320 nm for UVB intensity and from 280-400 nm for UVA+UVB intensity for each 30-minute time point and then found the average UVB and UVA+UVB intensity for the whole day including time when the sun was not shining.

**DNA extraction modifications**

We added 850 mL of modified lysis buffer [2], 100 µL of SDS (final concentration (C_f_)= 1%), and 100 µL of proteinase K (Qiagen, Valencia, CA) (C_f_ = 1 mg/ml) to each filter and incubated at 56^o^C for 14-16 hours. We then added 1 ml of Buffer AL (Qiagen, Valencia, CA) and incubated at 56^o^C for another 10 minutes. After the second incubation, we added 1 ml of 100% molecular grade ethanol and vortexed. We used a QIAvac 24 Plus (Qiagen, Valencia, CA) vacuum manifold to load the 3 ml of lysate in the spin columns. We then followed the DNeasy Blood and Tissue Kit protocol for the rest of the extraction.

**Inhibition testing**

Test samples from multiple time points in the study were investigated for inhibition using serial dilutions using both qPCR and conventional PCR [3]. Samples were diluted in two series: 5-fold (1:5, 1:25) and 10-fold (1:10, 1:100). For each assay (qPCR and conventional PCR), we aimed to find the minimal dilution needed to dilute out inhibitors but maintain the greatest amount of template. For conventional PCR, this was assessed visually with gel electrophoresis using the product of the first PCR amplification (40 cycles) to determine the minimum dilution at which a band is seen. We then diluted all extracts at the minimum dilution for consistency. For qPCR, we compared the change in Ct values for each set of dilutions (5-fold and 10-fold) to the expected Ct change (log2(5) = 2.32 cycles for 5-fold and log2(10) = 3.32 cycles for 10-fold dilution), assuming 100% efficiency. If the change for each set of dilutions was +/- 0.5 cycles than the expected change, we considered the sample inhibited.

**Bioinformatic processing**

In the Unix shell script, paired end reads were merged, reads were quality filtered, sequences were demulitplexed, primers were removed, and sequences were clustered into OTUs. We used PEAR (v0.9.6) [4] to merge paired end reads with the following parameters: maximum assembly length = 251, minimum assembly length = 150, quality score threshold = 15, and p-value = 0.01. Reads were filtered using the fastq_filter command in USEARCH (v1.8.0) [5] with the following parameters: minimum sequence length = 251, expected errors per read = 0.5. Sequences were demultiplexed; sequences were only retained if the tag added during amplification was found on both the forward and reverse read. We used cutadapt (v1.8.3) to remove primers. Singleton reads were removed. Sequences were clustered into OTUs by SWARM (v2.1.5) [6] with a cluster radius of 1; only OTUs with greater than 0.005% abundance were retained [7].

**References**

1. Maraccini PA, Mattioli MCM, Sassoubre LM, Cao Y, Griffith JF, Ervin JS, et al. Solar Inactivation of Enterococci and Escherichia coliin Natural Waters: Effects of Water Absorbance and Depth. Environ Sci Technol. 2016;50: 5068–5076. doi:10.1021/acs.est.6b00505

2. Bostrom KH, Simu K, Hagstrom A, Riemann L. Optimization of DNA extraction for quantitative marine bacterioplankton community analysis. Limnology and Oceanography Methods. 2004;2: 365–373.

3. Cao Y, Griffith JF, Dorevitch S, Weisberg SB. Effectiveness of qPCR permutations, internal controls and dilution as means for minimizing the impact of inhibition while measuring Enterococcus in environmental waters. J Appl Microbiol. 2012;113: 66–75. doi:10.1111/j.1365-2672.2012.05305.x

4. Zhang J, Kobert K, Flouri T, Stamatakis A. PEAR: a fast and accurate Illumina Paired-End reAd mergeR. Bioinformatics. 2014;30: 614–620. doi:10.1093/bioinformatics/btt593

5. Edgar RC. Search and clustering orders of magnitude faster than BLAST. Bioinformatics. 2010;26: 2460–2461. doi:10.1093/bioinformatics/btq461

6. Mahe F, Rognes T, Quince C, De Vargas C, Dunthorn M. Swarm: robust and fast clustering method for amplicon-based studies. PeerJ. 2014;2: e593–13. doi:10.7717/peerj.593

7. Bokulich NA, Subramanian S, Faith JJ, Gevers D, Gordon JI, Knight R, et al. Quality-filtering vastly improves diversity estimates from Illumina amplicon sequencing. Nat Meth. 2012;10: 57–59. doi:10.1038/nmeth.2276
