# Supplementary material for: Persistence of marine fish environmental DNA and the influence of sunlight
Source: PLoS One. 2017 Sep 15;12(9):e0185043. doi: 10.1371/journal.pone.0185043 (PMC5600408; doi:10.1371/journal.pone.0185043)
Supplement: S2 Text — Results of inhibition testing for qPCR and conventional PCR, presence/absence of species-level annotations over the course of the experiment. (DOCX) [file pone.0185043.s002.docx]

**S2 Text. Results and discussion supplement.**

**Results of inhibition testing for qPCR and conventional PCR**

We chose the following samples to test for inhibition in the conventional PCR assay: T0-A, T2-SA, T2-DA, T7-SA, T7-DA. PCR products from each sample/dilution were visualized using gel electrophoresis and scored for presence (+) or absence (-) of a band (Table S2.1). It was clear that inhibition was present in the conventional PCR assay as none of the 1:1 or 1:5 samples showed a band after the first amplification. We chose to dilute extracts 1:10 even though one of the five samples tested (T2-SA) did not have a band because we did not want to dilute out the sample in the later time point samples when we expected less template than the earlier time points.

**Table S2.1. Presence (+) or absence (-) of band scoring to test inhibition of conventional PCR assay using Mi-Fish-U primers.**

| *Sample* \| **Dilution** | **1:1** | **1:5** | **1:10** | **1:25** | **1:100** |
| --- | --- | --- | --- | --- | --- |
| *T0-A* | - | - | + | + | + |
| *T2-SA* | - | - | - | + | + |
| *T2-DA* | - | - | + | - | + |
| *T7-SA* | - | N/A | + | + | N/A |
| *T7-DA* | - | N/A | + | + | N/A |

“N/A” indicates sample was not run at that dilution

For the qPCR assay for *S. japonicus*, we tested the following samples for inhibition: T0-A, T1-SA, T4-SA, T5-SA. We ran all samples/dilutions in triplicate and used the average Ct value of the three triplicates to calculate the change in Ct between dilutions. The 1:100 dilutions of T1-SA and T4-SA were not assigned Ct values and we therefore could not compare 1:10 to 1:100 Ct changes. All of the samples tested for inhibition in the qPCR assay were within the range of expected change of Ct value (Table S2.2) and therefore were not considered inhibited.

**Table S2.2. Ct changes for 5-fold and 10-fold dilutions for testing qPCR assay inhibition.**

| **Dilution** | **1:1 to 1:5** | **1:5 to 1:25** | **1:1 to 1:10** | **1:10 to 1:100** |
| --- | --- | --- | --- | --- |
| *Sample* | **Expected Ct change: 2.32 (1.82/2.82)** | | **Expected Ct change: 3.32 (2.82/3.82)** | |
| *T0-A* | 2.39 | 2.33 | -- | -- |
| *T1-SA* | -- | -- | 3.04 | undetermined |
| *T4-SA* | -- | -- | 3.79 | undetermined |
| *T5-SA* | 2.28 | 2.13 | -- | -- |

“--” indicates sample was not run at that dilution

“undetermined” means no Ct value was assigned at that dilution.

**Presence/absence of species level annotations over the course of the experiment**

Considering OTUs annotated to the species level, just 0.36% of the total rarified sequence reads are represented in 12 different species. Similar to the results at the genus level, some species are present in only the surface or depth samples (e.g., *Oxyjulis californica*), some are found at both depths (e.g., *Triakis semifasciata*), and some have mixed results (e.g., *Sebastes babcocki*) (S3 Fig). Of the 12 species-level detections, 6 belong to the genera *Sardinops*, *Scomber*, or *Sebastes*, which is consistent with the dominance of these three genera in the environmental samples. The species level results are similar to the genus level results with only a portion of species present at T_0_; only 4 of the 12 species were detected at T_0_ but 10 of the 12 were detected at T_1_.

We used a GEE to investigate whether the presence of species depended on the time since the start of the experiment, sampling depth of the experiment, or their interaction (whether the impact of time differed by sampling depth) (S7 Table). We found no evidence to suggest that detection of species depended on sampling depth at time zero ($\beta$ = 0.17, p > 0.05). The interaction term between depth and time was not significant ($\beta$ = -0.12, p > 0.05), which means that sampling depth did not statistically affect the rate of species disappearance. Presence of species was negatively associated with time for samples ($\beta$ = -0.74, p < 0.05); the corresponding odds ratios were 0.48 for surface and 0.42 for depth, indicating that the odds of genera being present decreased with an increase in time.
