# Supplementary figures and images for: Persistence of marine fish environmental DNA and the influence of sunlight
Source: PLoS One. 2017 Sep 15;12(9):e0185043. doi: 10.1371/journal.pone.0185043 (PMC5600408; doi:10.1371/journal.pone.0185043)

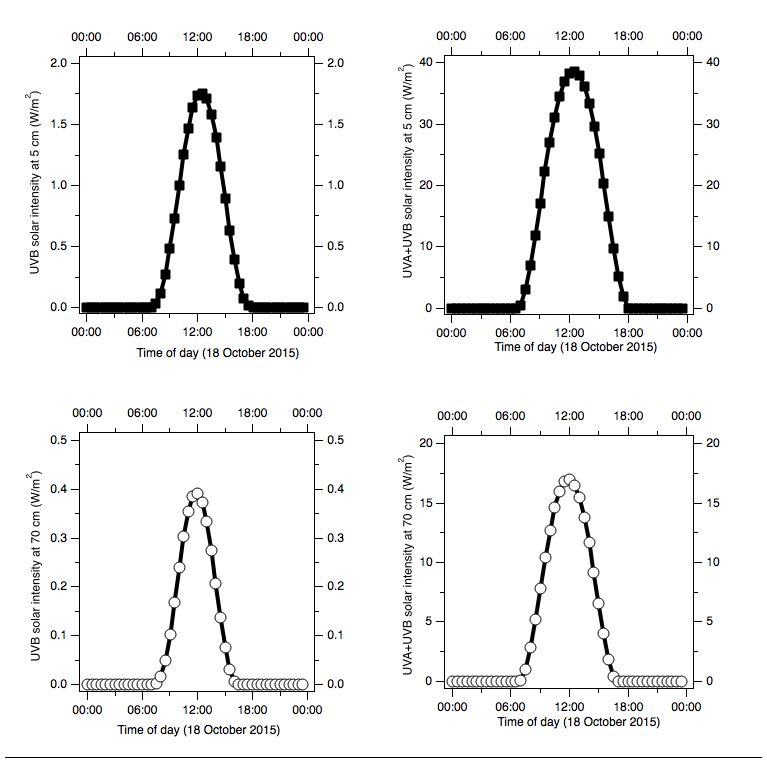

Supplement: S1 Fig — Top panels are at 5 cm below water surface (black squares), bottom panels are at 70 cm below water surface (open circles). Left panels only account for UVB solar intensity (W/m2), right panels account for UVA+UVB solar intensity (W/m2). (TIFF) [file pone.0185043.s003.tiff]

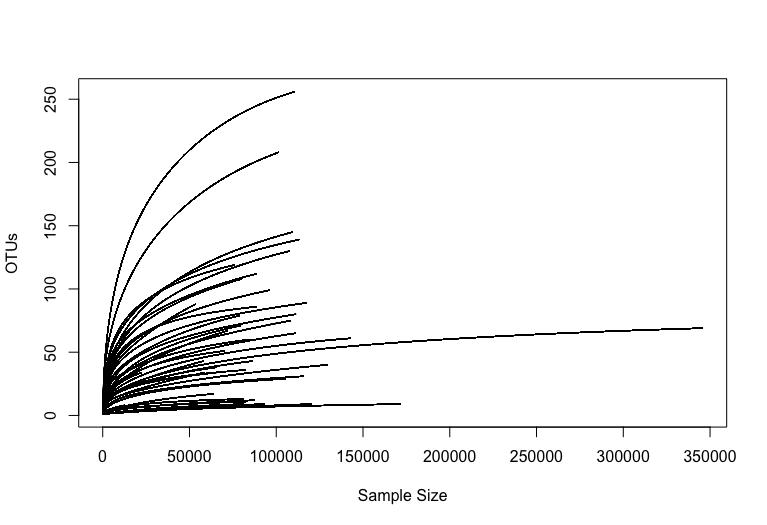

Supplement: S2 Fig — Y-axis shows number of unique OTUs identified and x-axis shows number of reads included in the sample size. (TIFF) [file pone.0185043.s004.tiff]

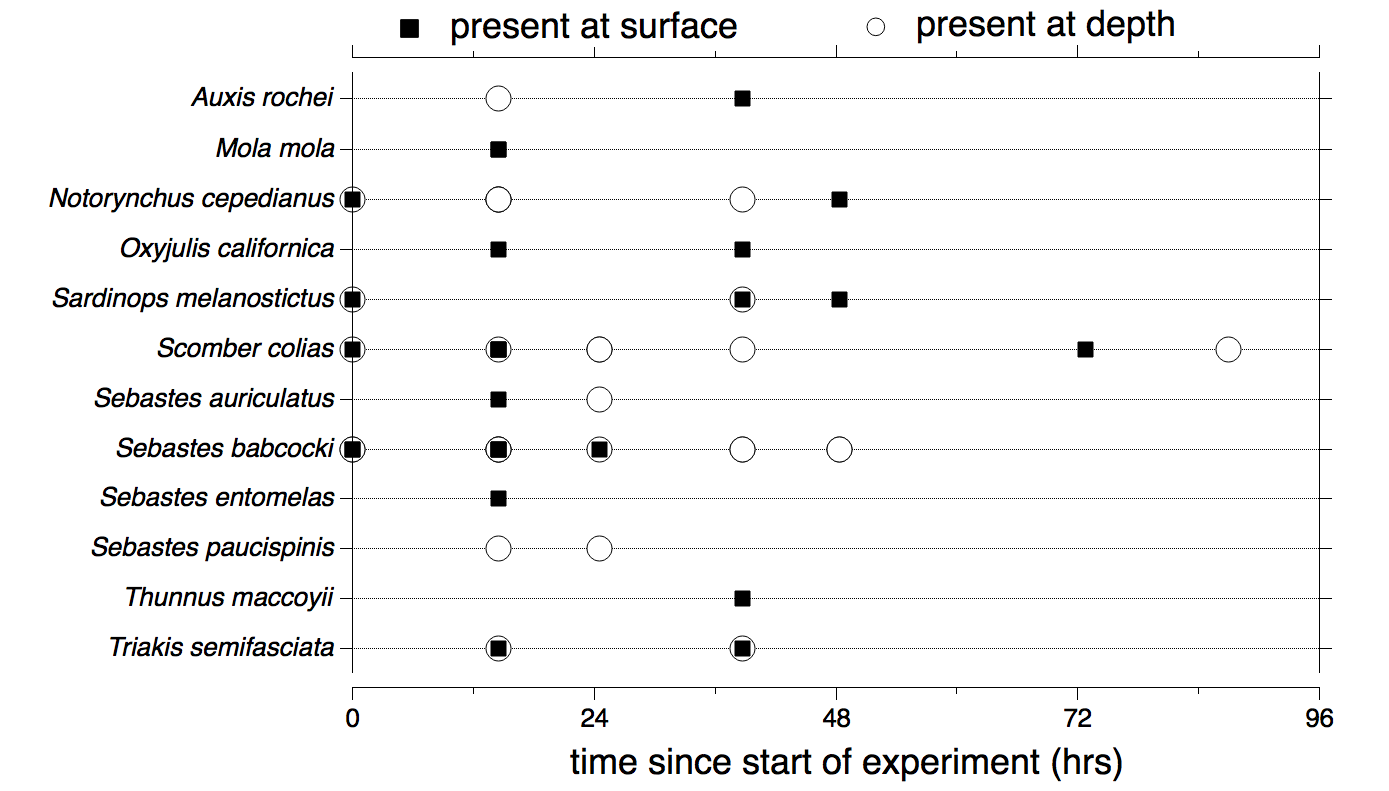

Supplement: S3 Fig — Solid squares indicate presence of the species in at least 1 biological replicate from surface samples; open circles indicate presence of the species in at least 1 biological replicate from depth samples. (TIFF) [file pone.0185043.s005.tiff]
